# Knowledge, attitudes, and practices of Hong Kong population towards human A/H7N9 influenza pandemic preparedness, China, 2014

**DOI:** 10.1186/s12889-015-2245-9

**Published:** 2015-09-22

**Authors:** Emily YY Chan, Calvin KY Cheng, Greta Tam, Zhe Huang, Poyi Lee

**Affiliations:** Collaborating Centre for Oxford University and CUHK for Disaster and Medical Humanitarian Response, JC School of Public Health and Primary Care, Faculty of Medicine, The Chinese University of Hong Kong, Hong Kong, Hong Kong SAR

**Keywords:** Knowledge, Attitude, Practice, Influenza A/H7N9, Pandemic preparedness, Chinese community, Hong Kong

## Abstract

**Background:**

Since SARS epidemic in 2003, Hong Kong has experienced several major epidemic risks, but how general community might react to the repeated infectious diseases health risks have not been studied. In 2013, imported human H7N9 influenza infected cases from China were reported. Our study aims to assess the knowledge, attitude and practice (KAP) concerning A/H7N9 among Hong Kong general population regarding pandemic preparedness in early 2014.

**Methods:**

A cross-sectional, population-based telephone survey study was conducted among the Cantonese-speaking population aged over 15 years in Hong Kong in February 2014. The study survey was composed of 78 KAP questions. Factors associated with individual and household pandemic preparedness were analyzed.

**Results:**

Final study sample was 1,020 with a response rate of 45.9 %. Among the respondents, most of them believed personal hygiene and avoidance of avian contacts were effective in preventing H7N9 infections. The majority of respondents had satisfactory hand hygiene practices and avoided touching avian species but did not employ other preventive measures. Female, 25 years old or older, white collar workers, people with chronic diseases and people living in the city center tended to report better hygiene practices. The average State-Trait Anxiety Inventory score was 1.85, similar to that of the period during the first wave and at the start of the second wave of the H7N9 epidemic. Self-reported face masks wearing when having influenza-like illness in general population dropped from 92.4 % during H5N1 period in 2007 to 39.0 % in this study.

**Conclusion:**

Hong Kong citizens show a low level of anxiety, misconceptions regarding the novel strains as well as gaps between perceived usefulness and practice of preventive measures towards influenza outbreaks. Educational campaigns and framing the issue to increase public and media awareness are crucial in preventing the current public fatigue towards outbreaks.

## Background

The experience of SARS coronavirus epidemic in 2003 has radically changed the concept and catalyzed actions globally and locally for combating infectious diseases outbreaks at the community level [1, 2]. Different health agencies globally are now striving to increase community resilience in combating this emerging infectious disease challenge. In particular, in the Hong Kong Special Administration Region of China, where a number of infectious diseases epidemic originated, resources were invested for enhancing infrastructure, policies and health education for pandemic preparedness and infection control. Whilst population resilience level in Hong Kong in handling infectious disease challenges are thought to be higher than other global cities, the mild clinical outcome of A/H1N1 influenza pandemic in 2009 and the limited transmissibility of the A/H5N1 avian influenza outbreaks in humans in 2007 may have modified citizens’ attitude and perceived risk towards similar outbreak incidences [3–5], and may have led them to underestimate the emerging A/H7N9 outbreak at the community level. A study conducted between 2006 and 2010 suggested that prolonged warning of a future pandemic was likely to cause pandemic fatigue in the public [6, 7]. For example, studies showed a decrease in self-reported mask use in case of influenza-like illness symptoms since the SARS in 2003 and post-SARS period [8]. There is currently limited literature to examine how prolonged warning may affect community perception and response to these large scale infectious disease risks.

**Fig. 1:**
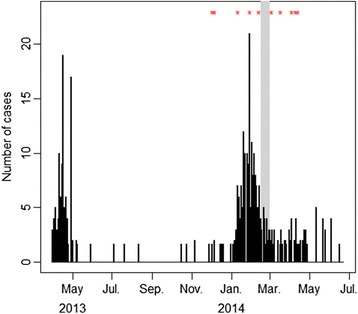
Timeline of laboratory-confirmed cases of influenza A(H7N9). Laboratory-confirmed cases of human infection with avian influenza A(H7N9) virus from 31 March 2013 to 23 June 2014, by date of notification to the World Health Organisation. Each asterisk represented an imported case reported in Hong Kong. The grey period on the right indicated the survey period

In March 2013, a human infected H7N9 influenza A virus (A/H7N9) case was first identified in Eastern China and caught global attention [9, 10]. Serious response level of the Preparedness Plan for Influenza Pandemic was activated in Hong Kong in Dec 2013 [11]. As of August 2014, a total of 450 confirmed cases were reported in various provinces in China [12]. A recent study in Hong Kong during the first wave of H7N9 epidemic in April 2013 showed low population anxiety levels [13]. Although current evidence of sustained human-to-human transmission for A/H7N9 is rare, a small cluster of infected cases within the family showed hospitalization might be required [14–16]. Confirmed case fatality rate was around 20 %, although the estimated symptomatic case fatality risk was lower [17]. At that time, there were a substantial number of confirmed cases of human infected cases of H7N9 avian influenza during the second wave of the epidemic (Fig. 1). Meanwhile, the infection rate of seasonal influenza was high in Hong Kong, as reflected by the Government Center for Health protection sentinel surveillance system [4].

Some personal behaviors had been suggested to be effective to control pandemics such as SARS and H5N1 in Hong Kong. Previous studies suggested that close contact with live poultry, visiting public places and the places having confirmed cases were risk factors, while frequent mask use, hand washing, disinfection, and avoidance of eating poultry were protective factors [13, 18, 19]. In this study, we conducted a representative, population-based telephone survey in Hong Kong 1) to investigate the current situation regarding the self-reported knowledge, attitude and practice of pandemic responses at individual level on the second wave human infected A/H7N9 avian influenza outbreak; and 2) to investigate factors associated with the self-reported personal hygiene practices.

## Methods

### Study design and study population

A cross-sectional, population-based telephone survey was conducted from 15–28 February 2014 in Hong Kong. The study population was the population aged 15 years or above residing in Hong Kong, including residents holding valid work or study visas. Exclusion criteria included i) non-Cantonese-speaking respondents; ii) overseas visitors holding tourist visas to Hong Kong; iii) 2-way permit holders from mainland China; and iv) those who were unable to be interviewed due to medical reasons.

### Instrument

A structured Chinese questionnaire was constructed and used for data collection. The questionnaire consisted of 78 closed ended questions that aimed to collect the following information from the respondents:

1. Socio-demographic and background information, including age, gender, district of residence, occupation and employment status, educational attainment, type and size of housing, and household income (total 21 questions).
2. Knowledge of H7N9 influenza A virus, including the differences between H7N9 and other respiratory diseases, transmission routes and “whether seasonal influenza vaccination could protect people from H7N9 infection” (total 9 questions).
3. Attitudes and risk perception towards H7N9 influenza A infections, including attitude towards preventive measures (total 9 questions), perception of H7N9 impact, perceived severity and infectivity, channel preference for information reporting and seeking, and their current anxiety level concerning the outbreak (total 30 questions). The 6 questions short form of the State-Trait Anxiety Inventory (STAI) was used for measuring anxiety [20]. A five point Likert-type scale were used to ascertain level of agreement or disagreement for the questions (from 1 to 5, 1 = strongly disagree, 2 = disagree, 3 = uncertain, 4 = agree, 5 = strongly agree).
4. Practices of preventive measures against H7N9 influenza A infections, including personal hygiene practices, avoidance of contact with avian species, household preparedness and vaccination practice (total 18 questions). A four point Likert-type scale were used to ascertain level of the practices (from 1 to 4, 1 = always, 2 = often, 3 = sometimes, 4 = never).
5. Active personal hygiene practice was defined by practicing 6 or more of the following 9 personal hygiene measures, including wash more hands, wash hands with soap, do not share utensils, wear mask when sick, bring own utensils during meals, avoid going to public places and avoid using public transport, avoid contacting with live poultry, avoid eating poultry and avoid going to places that had H7N9 confirmed cases. Active personal hygiene attitude was defined by regarding 6 or more of the above 9 personal hygiene measures above as useful for prevention H7N9.

Each interview took approximate 15–25 min to complete. The survey questionnaire was pilot tested in January 2014 (n = 50) to ensure practicability, validity, and interpretability of answers. The questionnaire was slightly refined for wording and format before distribution to the study sample based on the results of the pilot study. Results about vaccination practice and channels for disease surveillance have been reported elsewhere and not included in the analysis.

### Data collection

Randomly generated telephone numbers from a list of all land-based telephone numbers in Hong Kong was used as the sample frame [3, 8]. The telephone interviews were conducted by trained interviewers. The telephone calls were made in the evening on weekdays (6 pm-10 pm) and during daytime and evening on weekends (10 am-10 pm) to prevent over representation of the unemployed population. The subjects undergoing the interview were chosen based on the “last birthday method” [21, 22], in which the household member who was present in the household during the survey and whose last birthday was closest to the interview date was invited to participate. The subjects were invited on the basis of the proportion of age, gender and living district from the 2011 Hong Kong Population Census data. The sampling would continue until the quotas for each stratum were met. If the selected participant was busy or not there, up to 4 follow-up calls would be made. The numbers were called for a maximum of 5 times before being classified as unanswered.

### Statistical analysis

Differences in proportions between demographic characteristics in this survey and the Hong Kong Population Census data in 2011 were examined. Descriptive statistics for knowledge, attitude and practice of H7N9 influenza A prevention in the study population were calculated. Differences in proportions between groups were tested using Pearson’s Chi-square test or Fisher’s exact test. Likert-type scale results were collapsed to binary outcomes for analysis. Cut off point for questions with 5 point scales were defined as >3 and for questions with 4 point scale as >2. Univariate analysis was first conducted to identify apparent associations between individual pandemic preparedness practices with socio-demographic factors, perceived health risk, knowledge and attitudes towards A/H7N9 influenza virus. Backward multivariable logistic regression analyses were then conducted to identify factors associated with actual pandemic preparedness. All variables were retained in the final model if they had P-value < 0.05. All statistical analyses were conducted using R (version 3.0.2, R Development Core Team 2011). Statistical significance was set at α = 0.05.

### Research ethics

This study was approved by the Survey and Behavioural Research Ethics Committee of The Chinese University of Hong Kong. Oral consent was obtained from each of the participant at the beginning of the study. All collected data were anonymous.

## Results

**Fig. 2:**
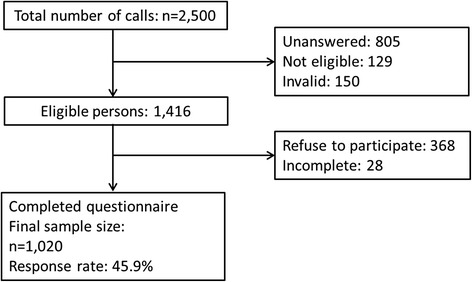
Study flow of the telephone survey

The final number of respondents who completed the survey was 1020, and the response rate was 45.9 % (1,020/2,221). Figure 2 detailed the data collection algorithm and response rate. Table 1 shows the socio-demographic characteristics of the study population compared with the general population in Hong Kong in 2011 [23].

**Table 1:** Socio-demographic characteristics of the respondents and the general population in Hong Kong in 2011

	Sample population		Hong Kong population 2011	Sample vs. census p-value^a^
	n	%	%	
Demographics				
Age (n = 1,020)				
15-24	143	14.0	14.0	0.99
25-44	348	34.1	35.5	
45-64	363	35.6	35.4	
≧65	166	16.3	15.1	
Gender (n = 1,020)				
Male	461	45.2	46.0	1.00
Female	559	54.8	54.0	
Education (n = 1,019)				
Primary education or below	138	13.5	22.7	0.18
Secondary education	517	50.7	50.0	
Post-secondary education(including diploma and certificate)	364	35.7	27.3	
Occupation (n = 1,006)				
White collar	411	40.9	NA	
Blue collar	96	9.5	NA	
Housewife, retired or unemployed	393	39.1	NA	
Students	106	10.5	NA	
Area of residence (n = 1,020)				
Hong Kong Island	185	18.1	18.0	1.00
Kowloon	308	30.2	29.8	
New Territories	527	51.7	52.2	
Marital status (n = 1,018)				
Single	355	34.9	42.2	0.36
Married	663	65.1	57.8	
Household income (n = 969)				
<$10,000	135	13.9	23.8	0.30
$10,000-19,999	220	22.7	23.8	
$20,000-39,999	346	35.7	29.0	
≧$40,000	268	27.7	23.5	
Type of housing (n = 1,017)				
Public housing	387	38.1	30.3	0.61^b^
Subsidized home ownership housing	160	15.7	15.9	
Private permanent housing	455	44.7	52.3	
Others	15	1.5	1.4	

^a^Chi-square test was used to measure the overall difference in proportions between this survey and the 2011 Hong Kong Population Census data. P-value < 0.05 indicates significant difference

^b^Fisher-exact test p-value was used

### Knowledge of A/H7N9 avian influenza

Of the respondents, only 25.3 % thought that they had sufficient knowledge to manage the risks that H7N9 avian influenza brings to their health and security. Majority of the respondents (62.5 %) mistook H7N9 influenza A virus as the common seasonal influenza of the current year and many respondents did not demonstrate accurate knowledge of known transmission routes of H7N9. Only 29.0 % of respondents correctly stated H7N9 can be transmitted via animals but not by insects and human feces. More than one fourth (27.5 %) thought insects can be vector for transmission and more than half (53.9 %) thought human feces was a possible transmission route. Meanwhile more than half thought those seasonal influenza transmission routes (human saliva 50.5 %, air 64.2 %, direct hand contact 59.9 %, indirect hand contact 55.0 %) can also transmit H7N9 avian influenza (Table 2).

**Table 2:** Knowledge assessment questions regarding A/H7N9 avian influenza virus

Characteristics	Yes (%)	No (%)	Do not know (%)
Do you think H7N9 is the seasonal flu of this year? (n = 1,016)	637(62.5)	312(30.6)	67(7.0)
Do you think H7N9 avian influenza can be spread by? (n = 1,009)			
Droplets by people	515(50.5)	489(47.9)	15(1.5)
Air borne	655(64.2)	360(35.3)	5(0.5)
Direct hand contact	611(59.9)	400(39.2)	8(0.8)
Indirect hand contact (e.g. via door handle)	561(55.0)	455(44.6)	3(0.3)
Human faeces	550(53.9)	445(43.6)	25(2.5)
Animals	906(88.8)	104(10.2)	10(1.0)
Insects	281(27.5)	711(69.7)	27(2.6)
Do you think seasonal flu vaccination can protect you from H7N9 virus infections? (n = 1,019)	370(36.3)	614(60.2)	35(3.4)

### Attitudinal determinants towards the impact of A/H7N9

Around half (51.8 %) of the respondents regarded Hong Kong as susceptible to infectious diseases outbreaks. The majority (86.0 %) believed that H7N9 would not acquire the ability of human-to-human transmission within the current year. Most (72.0 %) of them thought the chances of getting infected by H7N9 within the current year were low or very low. Only a small portion believed the H7N9 outbreak will cause high or very high impact for their health (28.9 %) and economic status (17.0 %). Only 37.8 % believed H7N9 will have high or very high impact affecting society. Respondents believed that H7N9 spread was controllable in general. 26.6 % of respondents did not believe the spread of H7N9 could be prevented at government policy level, and around half (22.6 %) believed that the spread could not be prevented at household and individual level.

The average anxiety level among the respondents towards the current infectious disease outbreak was 1.85 (IQR: 1.33, 2.33) on a scale of 1 (“not anxious at all”) to 4 (“very anxious”), which was similar to the first wave of H7N9 in April 2013 and during the first imported case in Hong Kong in December 2013 [13].

### Attitude and practice towards A/H7N9 personal hygiene prevention

Overall, most of the respondents thought that active personal hygiene measures reduced the risk of influenza transmission: Washing hands (87.2 %), using soap for washing hands (88.5 %), not sharing utensils (83.9 %), wearing mask when having respiratory infections (94.4 %), bringing their own utensils during meals (62.0 %), and to a lesser extent, avoiding public places and using public transport (58.4 %). They also had active attitude towards avoiding contact with live poultry (93.1 %), avoiding travel to previous human H7N9 infected areas (80.9 %) and to a lesser extent, avoiding eating avian species (56.0 %) (Table 3).

**Table 3:** Perceived usefulness and practice of preventive measures against human A/H7N9 influenza infections

Control measures that can protect from A/H7N9 infections	Thought it was useful for prevention		Always or usually practicing currently		Attitude vs. Practice
	n	%	n	%	p-value
Personal hygiene practices					
Wash hands more	888	87.2	990	97.1	0.260^*^
Use soap to wash hands	900	88.5	740	72.6	0.003
Do not share utensils	852	83.9	466	45.9	<0.001
Wear mask when sick	961	94.4	386	39.0	0.576
Bring own utensils during meals	626	62.0	16	1.6	0.314^*^
Avoid going to public places and use public transport	592	58.4	71	7.0	<0.001
Avoid source of A/H7N9 virus					
Avoid contacting with live poultry	949	93.1	767	75.2	<0.001
Avoid eating poultry	568	56.0	183	17.9	<0.001
Avoid going the places having H7N9 confirmed cases	820	80.9	569	55.8	<0.001
Had taken at least 6 out of all 9 measures above	825	80.9	192	18.8	<0.001

^*^Fisher’s exact test

Differences between the two groups were tested by Chi-square or Fisher’s exact test. Statistical significant level was set to p < 0.05

While for the actual hygiene practice, most respondents did not practice well the above control measures as shown in Table 3. Not all measures in practice were dependent with the perceived usefulness, such as washing hands, wearing mask when sick and not sharing utensils during meals. Only hand hygiene (97.1 % and 72.6 %) and avoiding contact with live poultry (75.2 %) were widely practiced. More than half (61.0 %) did not wear or just occasionally wore masks when they had respiratory infections. More than half shared utensils (54.1 %), and very few (1.6 %) brought their own utensils during meals when outside home. Over 90 % would not avoid going to public places or using public transport. Around half (55.8 %) would still visit places that had confirmed H7N9 cases.

### Factors affecting personal hygiene attitude

Univariate analysis of different socio-demographic factors, attitudinal determinants towards H7N9 and anxiety level that were associated with active personal hygiene attitude (defined as perceiving 6 or more out of 9 measures above useful) was included in the multivariable analysis. Backward multiple logistic regression revealed female gender (OR = 1.51, 95 % CI: 1.08-2.10, P < 0.05), working in office environment (compared to “white collar”, OR for “blue collar” = 0.55, 95 % CI: 0.33-0.92, P < 0.05), and those with high anxiety level towards infectious disease outbreak (compared to “Very low (STAI < 1.5)”, OR for “Medium(STAI 2.00-2.49)” =1.64, 95 % CI: 1.04-2.58, P < 0.05) had active personal hygiene attitude (Table 4).

**Table 4:** Multiple logistic regression analysis of factors that associate with personal hygiene attitude

Characteristics	^a^Personal hygiene attitude		^b^COR (95 % CI)	^c^AOR (95 % CI)
	Inactive N (%)	Active N (%)		
Gender				
Male	105(22.8)	356(77.2)	1	1
Female	90(16.1)	469(83.9)	1.54(1.12, 2.10)	1.51(1.08, 2.10)
Age				
15-24	20(14.0)	123(86.0)	1	
25-49	62(17.8)	286 (82.2)	0.75(0.43, 1.30)	
50-64	80(22.0)	283(78.0)	0.58(0.34, 0.98)	
≧65	33(19.9)	133(80.1)	0.66(0.36, 1.20)	
Occupation				
White collar	74(18.0)	337(82.0)	1	1
Blue collar	28(29.2)	68(70.8)	0.53(0.32, 0.89)	0.55(0.33, 0.92)
Housewife, retired or Unemployed	73(18.6)	320(81.4)	0.96(0.67, 1.38)	0.88(0.61, 1.27)
Student	16(15.1)	90(84.9)	1.24(0.69, 2.22)	1.22(0.67, 2.20)
Anxiety score				
Very low (STAI < 1.5)	61(22.0)	216(78.0)	1	1
Low (STAI 1.50-1.99	67(23.5)	218(76.5)	0.92(0.62, 1.36)	0.87(0.58, 1.30)
Medium (STAI 2.00-2.49)	39(14.4)	232(85.6)	1.68(1.08, 2.62)	1.64(1.04, 2.58)
High (STAI >2.49)	26(14.4)	155(85.6)	1.68(1.02, 2.78)	1.63(0.98, 2.72)
Believed Hong Kong is susceptible to infectious diseases outbreaks				
No	108(22.0)	383(78.0)	1	
Yes	87(16.5)	440(83.5)	1.43(1.04, 1.95)	
Believed that the spread cannot be prevented at household and individual level				
Not enough	112(21.9)	399(78.1)	1	
enough	83(16.4)	424(73.6)	1.43(1.05, 1.97)	

^a^Active personal hygiene attitude was defined by practicing 6 or more of the following 9 personal hygiene measures, including wash more hands, wash hands with soap, do not share utensils, wear mask when sick, bring own utensils during meals, avoid going to public places and avoid using public transport, avoid contacting with live poultry, avoid eating poultry and avoid going to places that had H7N9 confirmed cases

^b^COR, crude odd ratio in the univariate analysis

^c^AOR, Adjusted odd ratio in the multivariable analysis

### Factors affecting personal hygiene practice

Univariate analysis of different socio-demographic, attitude factors and active attitude towards preventive measures against H7N9 that were associated with active personal hygiene practice (defined as practicing 6 or more out of 9 measures above) was included in the multivariable analysis. Backward multiple logistic regression revealed female gender (OR = 2.17, 95 % CI: 1.48-3.18, P < 0.001), older age (compared to age 15–24, OR for age 25–49 = 4.81, 95 % CI: 1.37-16.89; OR for age 50–64 = 4.46, 95 % CI: 1.25-15.95; OR for age 65 or above = 6.19, 95 % CI: 1.60-23.99. P < 0.05 for all three), working in office environment (compared to “white collar”, OR for “blue collar” = 0.27, 95 % CI: 0.11-0.68, P < 0.01), having chronic diseases (OR = 1.84, 95 % CI: 1.20-2.82, P < 0.01) and living in city center districts (compared to suburb district, OR for densely populated district = 1.59, 95 % CI: 1.06-2.37, P < 0.05; OR for wealthy district = 2.58, 95 % CI: 1.65-4.05, P < 0.001) had better personal hygiene practices.

In terms of attitudinal factors, better personal hygiene practices were associated with those who had active attitude towards personal hygiene practices (OR = 2.83, 95 % CI: 1.60-5.01, P < 0.001), those with higher anxiety level towards infectious disease outbreak (compared to “Very low (STAI < 1.5)”, OR for “High (STAI > 2.49)” = 1.73, 95 % CI: 1.03-2.92, P < 0.05), those who believed H7N9 will obtain human to human transmission in the current year (OR = 1.85, 95 % CI: 1.17-2.93, P < 0.01), those who thought H7N9 will have higher impact to the Hong Kong society (OR = 1.67, 95 % CI: 1.16-2.41, P < 0.01), and those who thought they had sufficient knowledge to manage H7N9 risk (OR = 1.68, 95 % CI: 1.17-2.43, P < 0.01) (Table 5).

**Table 5:** Multiple logistic regression analysis of factors that associate with personal hygiene practice

Characteristics	^a^Personal hygiene practice		^c^COR (95 % CI)	^d^AOR (95 % CI)
	Inactive N (%)	Active N (%)		
Gender				
Male	402(87.2)	59(12.8)	1	1
Female	426(76.2)	133(32.8)	2.13(1.52, 2.98)	2.17(1.48, 3.18)
Age				
15-24	131(91.6)	12(8.4)	1	1
25-49	281(80.8)	67(19.3)	2.60(1.36, 4.98)	4.81(1.37, 16.89)
50-64	293(80.7)	70(19.3)	2.61(1.37, 4.98)	4.46(1.25, 15.95)
≧65	123(74.1)	43(25.9)	3.82(1.92, 7.58)	6.19(1.60, 23.99)
Marital status				
Single	305(85.9)	50(14.1)	1	
Married	521(78.6)	142(21.4)	1.66(1.17, 2.36)	
Occupation				
White collar	332(80.8)	79(19.2)	1	1
Blue collar	90(93.8)	6(6.3)	0.28(0.12, 0.66)	0.27(0.11, 0.68)
Housewife, retired or Unemployed	300(76.3)	93(23.7)	1.30(0.93, 1.83)	0.82(0.52, 1.29)
Student	95(89.6)	11(10.4)	0.49(0.25, 0.95)	1.97(0.53, 7.29)
Religion				
No	547(83.6)	107(16.4)	1	
Yes	280(76.7)	85(23.3)	1.55(1.13, 2.14)	
Chronic disease				
No	679(83.5)	134(16.5)	1	1
Yes	148(71.8)	58(28.2)	1.99(1.39, 2.83)	1.84(1.20, 2.82)
Household type				
Public housing	322(83.2)	65(16.8)	1	
Subsidized home ownership housing	139(86.9)	21(13.1	0.75(0.44, 1.27)	
Private permanent housing	351(77.1)	104(22.9)	1.47(1.04, 2.07)	
Vulnerable members				
No	330(84.4)	61(15.6)	1	
Yes	497(79.1)	131(20.9)	1.43(1.02, 1.99)	
Area of residence				
Suburb Districts	448(85.0)	79(15.0)	1	1
Densely Populated Districts	247(80.2)	61(19.8)	1.40(0.97, 2.02)	1.59(1.06, 2.37)
Wealthy Districts	133(71.9)	52(28.1)	2.22(1.49, 3.31)	2.58(1.65, 4.05)
^b^Attitude towards hygiene practice				
Inactive	177(90.8)	18(9.2)	1	1
Active	651(78.9)	174(21.1)	2.63(1.57, 4.39)	2.83(1.60, 5.01)
Anxiety score				
Very low (STAI < 1.50)	234(84.5)	43(15.5)	1	1
Low (STAI 1.50-1.99	243(85.3)	42(14.7)	0.94(0.59, 1.49)	0.82(0.49, 1.34)
Medium (STAI 2.00-2.49)	216(80.0)	55(20.0)	1.39(0.89, 2.15)	1.19(0.73, 1.94)
High (STAI >2.49)	131(72.4)	50(27.6)	2.08(1.31, 3.29)	1.73(1.03, 2.92)
Believed Hong Kong is susceptible to infectious diseases outbreaks				
No	415(84.5)	76(15.5)	1	
Yes	412(78.2)	115(21.8)	1.52(1.11, 2.10)	
Believed H7N9 will be capable of human-to-human transmission this year				
Not likely	730(83.3)	146(16.7)	1	1
Likely	96(68.1)	45(31.9)	2.34(1.58, 3.48)	1.85(1.17, 2.93)
Have very high chance to be infected by H7N9				
Not likely	777(82.0)	171(18.0)	1	
Likely	51(71.8)	20(28.2)	1.78(1.04, 3.07)	
Impact of H7N9 affecting your health				
Low impact	620(83.4)	120(16.6)	1	
High impact	220(75.3)	72(24.7)	1.64(1.18, 2.28)	
Impact of H7N9 to Hong Kong				
Low impact	540(84.9)	96(15.1)	1	1
High impact	287(74.9)	96(25.1)	1.88(1.37, 2.58)	1.67(1.16, 2.41)
Perceived knowledge to combat H7N9 outbreak				
Not enough	602(84.0)	115(16.0)	1	1
Enough	225(74.8)	76(25.3)	1.77(1.27, 2.45)	1.68(1.17, 2.43)

^a^Active personal hygiene practice was defined by practicing 6 or more of the following 9 personal hygiene measures, including wash more hands, wash hands with soap, do not share utensils, wear mask when sick, bring own utensils during meals, avoid going to public places and avoid using public transport, avoid contacting with live poultry, avoid eating poultry and avoid going to places that had H7N9 confirmed cases

^b^Active personal hygiene attitude was defined by regarding 6 or more of the above 9 personal hygiene measures above as useful for prevention H7N9

^c^COR, crude odd ratio in the univariate analysis

^d^AOR, Adjusted odd ratio in the multivariable analysis

## Discussion

We assessed the knowledge, attitude and actual practice of the Hong Kong population for pandemic preparedness during the second wave of human infected H7N9 epidemic in mainland China and when the seasonal influenza activity was high in the community. The result showed respondents still harbored misconceptions of H7N9 influenza, were generally not anxious about the H7N9 outbreak, and were not sufficiently practicing protective measures against infections.

### Demographic patterns and knowledge gaps

Our study found several demographic determinants coincided with other studies in previous literature of respiratory epidemics and pandemics [24]. Female, 25 years old or older, having chronic disease, living in city center district and white collar workers were associated with better preventive behaviors.

Similar to other previous influenza studies [25, 26], respondents did not have detailed understanding of H7N9 avian influenza. A lot of them mixed up A/H7N9 with other types of influenza or other respiratory virus. Mixing up the characteristics may lead to inappropriate health behaviors, making the protection suboptimal or even risky. Although some precautionary measures are similar for preventing H7N9 influenza and other respiratory infections, specific precautions against avian types of influenza, in particular those avoiding contacts with live poultries and their feces should be emphasized to increase individual awareness for better informed protective measures.

### Population anxiety and risk perception

Our study was conducted during the second wave of H7N9 epidemic, at the time, Hong Kong had 5 imported cases with high case fatality ratio, during the winter peak of seasonal influenza. However we found the population still did not take H7N9 seriously. This was reflected in their attitudinal determinants towards the impact of A/H7N9 and their low anxiety level (STAI of 1.85). The population anxiety level was similar to a recent study in Hong Kong during the first wave of H7N9 epidemic in April 2013, and did not increase comparing to the beginning of the second wave when the first case imported in Hong Kong in December 2013 [13]. The anxiety score was also similar to that during the first wave of H1N1 in 2009. In contrast, the anxiety score was much higher (STAI 2.50) during SARS [5]. 74.8 % of Hong Kong population would avoid going to crowded places, and 74.7 % and 71.8 % would avoid going to mainland China and hospitals respectively during SARS period in 2003 [27]. In current study, only 7 % would avoid going to public places and using public transport, and 55.8 % avoid going to places having H7N9 confirmed cases. The findings may indicate public fatigue in response to prolonged warning of influenza pandemic, with people reporting less adoption of infection control behaviours. Though low anxiety level is expected, the misconceptions, inactive attitudes and lack of preventive practices are not optimal for preparedness of future epidemics.

### Gaps between perceived usefulness and practice of preventive measures

There are significant gaps between perceived usefulness and practice of mask wearing and do not share utensils during meals. The population showed high compliance with hand washing. 94 % of the Hong Kong population believed that hand washing was efficacious in preventing human-to-human avian influenza during H5N1 period [19] while 97.8 % perceived washing hands frequently as an effective public health measure during H1N1 period [8]. In the current study, this figure was 87.2 %. This might be due to public health announcements on mass media, emphasizing the importance of washing hands frequently. In contrast, for mask wearing and utensils sharing, the proportion of actual practices was far lower than perceived usefulness. 92.4 % of the Hong Kong residents wore face masks when having influenza-like illness during H5N1 period in 2007 [19] and 88.7 % in 2009 during H1N1 period [8]. In the current study, it dropped to 39.0 %. Barriers for taking action may be partly due to discomfort when wearing mask, traditional Chinese norm of sharing utensils when dining, and the inconvenience. Nevertheless, our study results showed the main reason hindering individual control behaviors were that people did not consider H7N9 outbreak a serious threat. Their attitude could be reflected by their low perceived severity and infectivity of the virus. Targeted interventions may focus on increasing the awareness of the population towards the outbreak according to current situations. And further analysis will be needed to identify other specific barriers and to increase the self- efficacy in overcoming them, thus changing the health behavior for better protection.

### Limitations

Limitations in the study included the methodological limitations of telephone survey. Firstly, households with no possession of a land-based telephone service may be missed. Nonetheless, the penetration rate of residential fixed line service in Hong Kong was 102.6 % in November 2013. The residential fixed line penetration is calculated by dividing the number of residential fixed lines by the number of households in Hong Kong [28]. Almost all households have at least one home based telephone service in Hong Kong. Secondly, our sample population had higher household income and education level than the Hong Kong general population. This may overestimate the overall results as these groups are more knowledgeable and have better practices for pandemic preparedness in general. Thirdly, there may be some reporting bias. Data were self-reported and data from non-respondents could not be obtained.

Our results may not be generalizable to other countries or cities that have not experienced previous severe epidemics such as SARS. This study presents as a unique reference for pandemic preparedness behavioral response during a second wave epidemic in an urban setting. External factors may influence the stability of the responses during the survey period. Nevertheless, we managed to finish the field data collection within a short period (two weeks) to produce a stable response.

## Conclusion

Similar to previous studies, Hong Kong citizens show a low level of anxiety, misconceptions regarding the novel strains as well as gaps between perceived usefulness and practice of preventive measures towards influenza outbreaks. Educational campaigns and framing the issue to increase public and media awareness is crucial in preventing the current public fatigue towards outbreaks.

### Availability of data and materials

Not applicable.
